# Phenological Development, Productivity, and Oil Profiles of Different Safflower Cultivars for Biofuel Production

**DOI:** 10.1021/acsomega.5c02782

**Published:** 2025-11-17

**Authors:** Raimunda Adlany Dias da Silva, Luisa Gouveia, Thomaz Gabriel Barros da Rocha, Amanda Duarte Gondim, Juliana Espada Lichston, Nataly Albuquerque dos Santos

**Affiliations:** † Programa de Desenvolvimento e Meio Ambiente, (PRODEMA), 28097 Universidade Federal da Paraíba, 58051-900 João Pessoa, Brasil; ‡ 511948 Laboratório Nacional de Energia e Geologia − UBB − Unidade de Bioenergia e Biorrefinarias, Lisboa 1649-038, Portugal; § GreenCoLab, 22 8005-139 Faro, Portugal; ∥ Departamento de Ecologia-DECOL, Centro de Biociências, Universidade Federal do Rio Grande do Norte, 59078-900 Natal, Brazil; ⊥ Instituto de Química, Campus Central, Universidade Federal do Rio Grande do Norte, 59078-900 Natal, Brasil; # Laboratório de Investigação de Matrizes Vegetais energéticas, Departamento de Botânica e Zoologia, Universidade Federal do Rio Grande do Norte, 59078-900 Natal, Brasil; ∇ Departamento de Tecnologia de Alimentos, CTDR, Universidade Federal da Paraíba, 58051-900 João Pessoa, Brasil

## Abstract

The production of oilseed biomass to meet the demand of the energy sector is constrained by several factors, including regional soil and climate conditions, phenological and production issues, such as yield and oil profile, and the compatibility of these factors with the requirements of the energy sector. Safflower is a small oilseed, and its brief phenological cycle and high productivity, concentration, and oil profile distinguish it as a notable candidate for research on energy applications. The objective of this study was to analyze the germination, seed vigor, yield, and oil profile parameters of safflower cultivars (IMAmt 1470, IMAmt 894, and IMAmt S525) with a view to determining their potential as biomass for the biofuel production chain, especially biodiesel and renewable aviation hydrocarbons. Safflower cultivars displayed high germination rates and germination vigor after 12 months of storage. They also met the production standards of 6797.7 kg ha^–1^ in 2021. The cultivar IMAmt-S525 exhibited a high oil content of 35%. The oil compositions of the safflower cultivars included in this study were found to be 9.7% palmitic acid (IMAmt1470), 71.82% linoleic acid (IMAmt 894), and 41% oleic acid (IMAmt 894 harvest 2022). It is recommended that the following cultivars be selected for production: IMAmt 894, IMAmt-S525, and IMAmt 1470, taking into consideration the physiological, production, and oil composition parameters. Since all three cultivars have high standards of physiological quality, productivity, and oil yield, they have the potential to be used as biomass to diversify oilseed matrices for biofuels.

## Graphical abstract

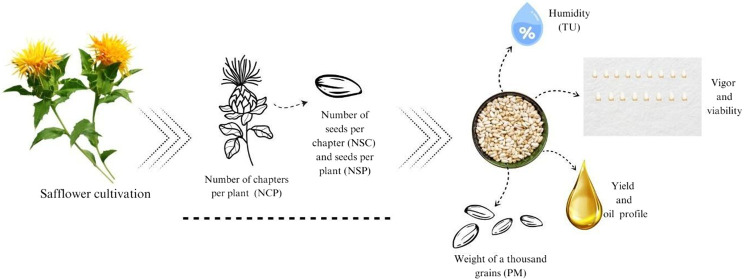

## 1 Introduction

Oilseed plants have been widely investigated as biomass sources for the production of biofuels such as biodiesel, and, recently, for the production of renewable hydrocarbons for aviation^1–5^ to mitigate greenhouse gas (GHG) emissions associated with the use of conventional energy sources. The air transport sector contributes 2% to global GHG emissions^5,7^ and has invested in the production of biofuels (biojet fuel) as an emissions mitigation strategy.^8^ Among its targets is the mandatory use of renewable fuels between 2027 and 2035, established in the international agreement, the Carbon Offsetting and Reduction Scheme for International Aviation—CORSIA/ICAO.^9^ The production of biofuels and hydrocarbons makes use of a range of biomass such as sugar,^10^ lignocellulosic material,^11^ and vegetable oils,^12^ with the application of diverse synthesis routes aligned with the physicochemical characteristics of the biomass. Biomasses must be adequate in their chemical and physical properties affected by basic and compositional characterization analyses.^13^

In this context, the production of oilseed biomass to meet this expected demand faces some drawbacks, such as soil and climatic, geo-environmental, and geospatial conditions.^6^ High temperatures and low water availability, for example, which are characteristics of semiarid environments, directly influence the development and productive aspects of oilseeds^14,15^ and are thus restrictive factors for plant production.^16,17^ The Brazilian semiarid region is characterized by low annual rainfall of 800 mm, high temperature, and evapotranspiration,^18,19^ which are limiting conditions for the development of oilseeds conventionally used for biofuel production. It is therefore imperative, given the need to supply oil biomass to the renewable energy sector, to diversify the list of oilseed-producing matrices. Potential crops for cultivation in semiarid environments that perform well in terms of morphophysiology, yield, oil content, and oil quality are the preferred raw materials for the production of aviation biofuels.^14^

In this context, *Carthamus tinctorius* L., safflower, a small oleaginous plant of the Asteraceae family, is characterized by its high resistance to low water supply and the high quality of its oil produced.^20^ The phenological cycle is short, between 130 and 150 days,^21,22^ with a reduction to 75 days under the soil and climate conditions of the Brazilian semiarid region.^23^ Seed yields can range from 1489 to 3704 kg ha^–1^ ^24^ and seed oil concentration from 20.78 ^25^ to 36.69%.^26^ The crop also contributes to low GHG emissions in its life cycle.^21^

The aim of this study was to analyze the production parameters, such as germination, yield, oil profile, and seed vigor during storage, of safflower cultivars (IMAmt 1470, IMAmt 894, and IMAmt S525) to assess their potential as biomass to diversify the biofuel production chain, especially for biodiesel and renewable aviation hydrocarbons in the semiarid region.

## 2 Methodology

### 2.1 Characterization of the Study Area and Field Cultivation

The three safflower cultivars, IMAmt 894, IMAmt S525, and IMAmt 1470, obtained from the Mato Grosso Institute of Cotton (IMAmt), were grown in an area of 3.456 m^2^ on the premises of the experimental field of the Federal Institute of Education, Science and Technology of Rio Grande do Norte (IFRN), located at the Apodi campus in Rio Grande do Norte, Brazil. The geographical coordinates of this site are 5°37′34.0″S 37°48′27.0″W, with an altitude of 141 m. The region’s climate is semiarid, with an average annual temperature of around 28.1 °C, with highs of 36 °C and lows of 21 °C. It has a relative humidity of 68% with 2700 h of sunshine annually, with a rainy season from March to May and a dry season between June and February.^27^

Sowing took place in a 3 × 3 design (Figure 1), three cultivars, and three replications, each lot with 37.5 m × 8 m, in an area of 144 m × 24 m, with the first cultivation cycle with sowing on June 29, 2021 and harvest on September 22nd, 2021 and the second cultivation cycle with sowing on August 02, 2022 and harvest on November 01, 2022. The spacings used were 40 cm between rows, 15 cm between holes, and 1.5 m between plots. The average depth of the seed sown was 2 cm. The irrigation system used was a drip irrigation system with a 30-minute rule duration and a flow rate of 1.6 L/h. Watering took place daily from sowing until 15 days after emergence, then every 2 days until 60 days after germination, and in the last 22 days of the development cycle, irrigation was suspended.

**Figure 1:**
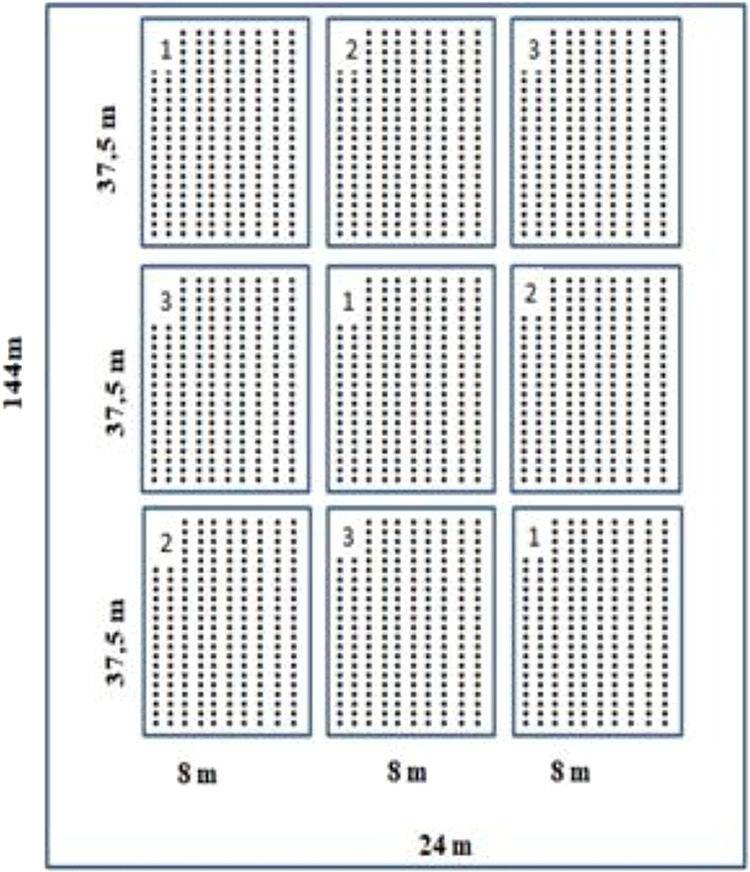
Experimental design of the field cultivation of the three safflower cultivars.

Soil fertility and correction for sunflower was done according to the recommendations of the Agricultural Research Company of Rio Grande do Norte EMPARN, based that used for sunflower, which belongs to the same botanical family as safflower, was followed. This involved applying 50 kg/ha of simple superphosphate (20% P_2_O_5_), 20 kg/ha of urea (45% N), and 20 kg/ha of potassium chloride (58% KCl), as recommended by Castro et al.^28^ for sunflower, which belongs to the same botanical family as safflower. A preliminary soil analysis was carried out, which showed the following characteristics: calcium = 4.40 cmolc/dm^3^, phosphorus = 16.3 mg/dm^3^, potassium = 187 mg/dm^3^, sodium = 76.3 mg/dm^3^, and pH = 7.78. The soil is classified as argisol soil.

The irrigation system used was a drip irrigation system with a 30-min rule duration and a flow rate of 1.6 L/h. Watering took place daily from sowing until 15 days after emergence, then every 2 days until 60 days after germination, and in the last 22 days of the development cycle, irrigation was suspended.

The selection of safflower cultivars was based on previous comparative studies. The cultivars with the best performance were retained, and a new cultivar was added for testing.

#### 2.1.1 Edaphoclimatic Conditions during the Experiment Period

Figure 2 shows the environmental conditions of humidity, temperature, sunshine, and rainfall during the cultivation of the different safflower cultivars in 2021­(A) and 2022­(B). Data on atmospheric conditions—average humidity (%), sunshine duration (h), maximum temperature (°C), minimum temperature (°C), and precipitation (mm)—were obtained from the website of the National Institute of Meteorology (INMET), a Brazilian government agency that uses a local weather monitoring station (CODE 82590).

**Figure 2:**
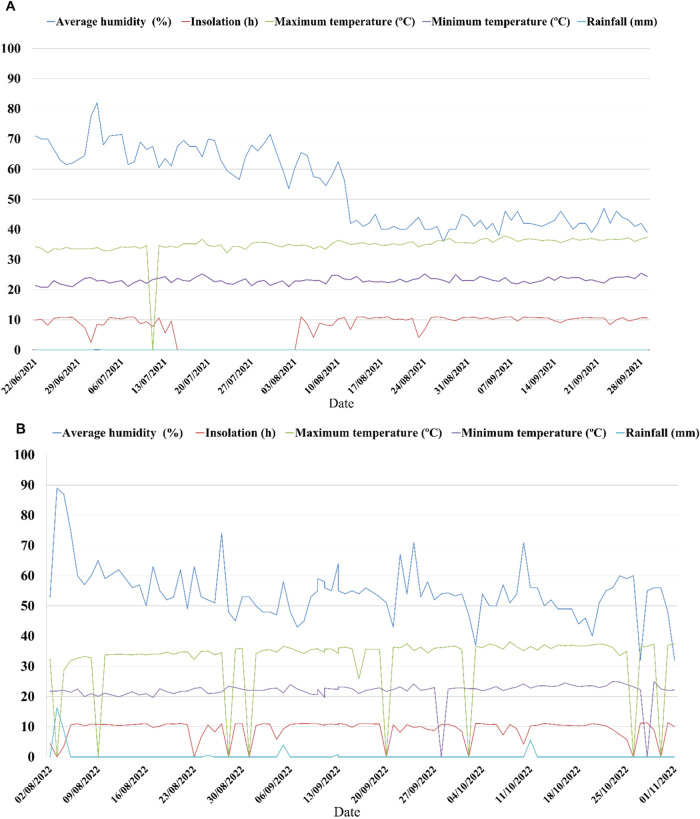
Climatic conditions during the cultivation cycle of safflower cultivars in 2021­(A) and 2022 (B).

### 2.2 Analysis of Germination Parameters during Storage

The seeds collected in 2021 were packed in 1 L PET bottles and stored at room temperature for 18 months. Germination was carried out on the freshly harvested seeds, and every 3 months after storage (0, 3, 6, 9, 12, 15, 18 months of storage), accounting for seven germination tests adapted from the rules of seed analysis in ref^29^. The photoperiod was 12/12 h light/dark, and the temperature was 34 °C, as indicated by Torabi et al.^30^ to be ideal for germination, in a BOD-type chamber. Every day, the number of germinated seeds was evaluated, the radicle was 2 mm long, and after 7 days of incubation, the final germination percentage and germination speed index were measured.^29,31^ These analyses were repeated every 3 months for a period of 18 months, totaling 6 analyses to check the viability and vigor of the three safflower cultivars in storage.

### 2.3 Moisture Content

The moisture content of the freshly harvested seeds was analyzed using an aluminum capsule, in which 4.5 ± 0.5 g of the sample was added and heated in an oven at a temperature of 130 °C, where it remained for a period of 24 h. Afterward, the weight was calculated using a precision scale, and the moisture content (TU%) was measured according to the following formula: $U\;\%=\frac{100\left(P-p\right)}{P-t}$,^29,32^ where *P* = initial weight (weight of the container and its lid plus the weight of the wet seed), *p* = final weight (weight of the container and its lid plus the weight of the dry seed), and *t* = tare weight (weight of the container with its lid).

### 2.4 Production Parameters

The number of chapters per plant (NCP), number of seeds per chapter (NSC), seeds per plant (NSP), and the weight of a thousand grains (PM) of the three safflower cultivars from the 2021 and 2022 crops were counted according to the rules for seed analysis (RAS)^29^ and those used by Araujo et al.^33^ The final yield was calculated using the parameters mentioned above and expressed in kg ha^–1^.

### 2.5 Yield and Oil Profile of the Three Safflower Cultivars

The cake obtained by grinding the seeds of each safflower crop (2021 and 2022 crops) was subjected to oil extraction using the Soxhlet system in an oil bath at 95 °C, with a cycle duration of 6 h and three repetitions. The organic solvent, 150 mL *n*-hexane (Anhydrol, purity 98.8%), and 10 g of sample mass were used. After extraction, the solvent was removed using a rotary evaporator to separate the oil, and the oil volume was expressed as a percentage.^23^ The amount of oil obtained was expressed as a percentage.

Subsequently, the oil from safflower cultivars from both crops was subjected to GC/MS gas chromatography using a Shimadzu apparatus with a 5% phenyl methylpolysiloxane column. For this, safflower oil was transesterified using 4 g of potassium hydroxide (KOH) in a molar ratio of 16:1 at a temperature of 60 °C for 4 h while stirring at 700 rpm^34^ as a pretreatment. GC/MS gas chromatography is used to analyze the fatty acid profile and determine the composition and proportion of saturated, monounsaturated, and polyunsaturated acids present in the oils

### 2.6 Statistical Analysis

The data on germination markers, moisture content, and yield parameters were subjected to two-way ANOVA, followed by the Tukey test (*p* < 0.5), comparing cultivars and years of cultivation.

## 3 Results and Discussion

### 3.1 Moisture Content (TU) and Weight of a Thousand Grains (PM) of the Three Safflower Cultivars

The crop’s phenological cycle lasted 86 days in 2021 and 92 days in 2022. In 2021, the average humidity was 53%, with an average of 10 h of sunshine, and the temperatures were 35 and 23 °C (maximum/minimum), with no precipitation. In 2022, the average humidity was 52%, with an average of 10 h of sunshine, and the temperatures were 36 and 23 °C (maximum/minimum), with no precipitation.

The cycle for the crop in the two years analyzed was longer by 10 days and 20 days, respectively, than that observed by da Silva et al.^23^ in a crop grown in the Brazilian semiarid region (72 days) in 2018, but shorter than that observed by Liccata et al.,^35^ who recorded a phenological cycle of 220 days for the crop.

When analyzing the seeds produced, one of the essential parameters is the moisture content, as it can directly affect the physiological quality, reflecting the decrease in their germination potential.^36^ Among the cultivars studied in 2021, the cultivar with the highest seed moisture content (TU) was IMAmt 894, with a percentage of 8.6%. When compared to the other cultivars, this is higher than that observed for cultivars IMAmt 1470 and IMAmt S525 (Table 1). In 2022, there was a variation in TU percentages between 5.6% for IMAmt S525, which had the lowest TU, and 7.1% for IMAmt-894, which had the highest TU (Table 1). There was a statistically significant variation between the cultivars and crop years. The 1% variation in atmospheric humidity in 2021 (54%) and a temperature of 1 °C in 2022 (36%) may have influenced the obtained results.

**Table 1:** Moisture Content in Seeds (TU, in %) and Weight of a Thousand Grains (PM, in g) of the Seeds of the Three Safflower Cultivars (IMAmt 1470, IMAmt 894, and IMAmt S525) from the 2021 and 2022 Harvests^a^

	humidity of seeds (TU)		grain weight (PM)	
cultivars	2021	2022	2021	2022
IMAmt 1470	8.2 ± 0.5 Aa	6.9 ± 0.5 Ab	41.7	39.25
IMAmt 894	8.6 ± 0.5 Bb	7.1 ± 0.5 Aa	33.3	40
IMAmt S525	7 ± 0.5 Ca	5.6 ± 0.5 Ab	31	36.6

a Lowercase letters compare different averages in the columns, different lowercase letters indicate *p* < 0.5, and different uppercase letters between the rows compare averages and indicate *p* < 0.5.

The TU of seeds at adequate levels is an essential factor, as water is involved in the metabolic and physiological processes of the seeds with enzymatic activation to start germination. Water plays a role in activating mitochondrial activity and producing energy to activate germination metabolism.^37^ Both high and low levels of TU cause damage to the seed development process, and each species has its most appropriate levels of TU, which promote the preservation of the viability of seeds.^38^

The moisture content of seeds of the safflower cultivars observed here is lower than that cited in the literature for soybeans,^36^ which indicates percentages between 12 and 14%, the main oilseed used in the biofuel industry in Brazil. This is close to the TU observed for peanut and sesame crops by Geetha et al.^39^ who recorded TU values of 8%. Lower contents have been reported in the literature between 8.5%,^40^ 8–10%,^41,42^ 9%,^43^ 6–8%,^44^ indicating that this TU range for safflower seeds was found in the batches with the best germination performance and germination speed. These criteria indicate the physiological quality of the seeds.^45^ The percentages cited in the literature for TU are similar to those observed here for safflower cultivars (Table 2). This may indicate the possible high physiological quality of the seeds of the safflower cultivars studied here.

**Table 2:** Seed Yield per Hectare (kg ha^–1^) (SPH) and the Number of Seeds per Plant (NSP) in 2021 and 2022 Harvests of the Three Safflower Cultivars IMAmt-1470, IMAmt-894, and IMAmt-S525^a^

	productivity, kg ha^–1^ (SPH)		seeds per plant (NSP)	
cultivars	2021	2022	2021	2022
IMAmt 1470	5.981,130 Aa	1.604,957 Bb	597,634 Aa	170,377 Bb
IMAmt 894	6.797,728 Aa	2.124,33Bb	846,812 Aa	221,315 Bb
IMAmt S525	5245,720 Ab	4406,053 Aa	705,069 Aa	501,600 Aa

a Lowercase letters comparing averages in columns indicate *P* < 0.5, and uppercase letters compare averages in rows.

Seed moisture content is one of the factors that can influence the weight of a thousand seeds (WE).^46^ Parameters can indicate seed lots with low physiological quality, when the MP is high or below the standard for the seed lot according to the species, as adequate levels of TU promote the preservation of seed viability.^37,38^ For thousand-grain weight (GK), the cultivar with the highest numerical value was IMAmt 1470 in the 2021 harvest and IMAmt 894 in the 2022 harvest (Table 1). The weight of a thousand grains (WG) in 2021 for the IMAmt 1470 cultivar was the highest, which was 8.4 g higher than that of the IMAmt 894 cultivar and 10.7 g higher than that of the IMAmt S525 cultivar.

In 2022, the cultivar with the highest PM was IMAmt 894 (40 g), followed by IMAmt 1470 and IMAmt S525 (Table 1). Higher values of between 9 and 10 g^47^ for the crop were observed when compared to those recorded here for cultivars IMAmt 1470, grown in 2021, and IMAmt 894, grown in 2022, with higher values obtained between cultivars and between crop cycles. A similar response in terms of changes in PM values between crop years was also reported by Manvelian et al.,^48^ who obtained values of around 34.8 g for the crop. Records close to those observed here for the crop were reported by Shahrokhnia et al.^49^ with a record of 28.7 g for the first year of cultivation and 27.8 g for the second year of cultivation, values lower than those observed in this study for safflower cultivars. This suggests that possible annual variations influence the crop in terms of both humidity (TU) and grain weight (PM).

In addition to the weight of a thousand grains (PM), which is related to the physiological quality of the seed batch, batches can be differentiated between those that are outside the standard for the species and cultivar, i.e., with WG values below or above those indicated in the literature^46^ and is used to plan experimental sowing designs. Seeds of better physiological quality provide greater germination success.^50^

### 3.2 Germination and Germination Speed Index of Seeds of the Three Safflower Cultivars

The germination percentage, in addition to being the first stage of development in plants, is used to indicate the vigor and viability of seeds.^51^ The final germination rate of the seeds produced in 2021 among the cultivars, IMAmt 1470, was the one that differed statistically from the other cultivars (*p* < 0.5), with the highest germination rate of 95%, followed by cultivar IMAmt S525 with 78%, and last cultivar IMAmt 894 with the lowest germination rate of 68%. However, in 2022, the IMAmt 894 cultivar showed the highest final germination percentage of 88%, followed by the IMAmt S525 cultivar with 80%, and finally the IMAmt 1470 with 75%, with a statistically significant difference (*p* < 0.5), with the morphoclimatic conditions in 2021 being favorable for the IMAmt 1470 cultivar, and those in 2022 favorable for the IMAmt 894 cultivar (Figure 3A).

**Figure 3:**
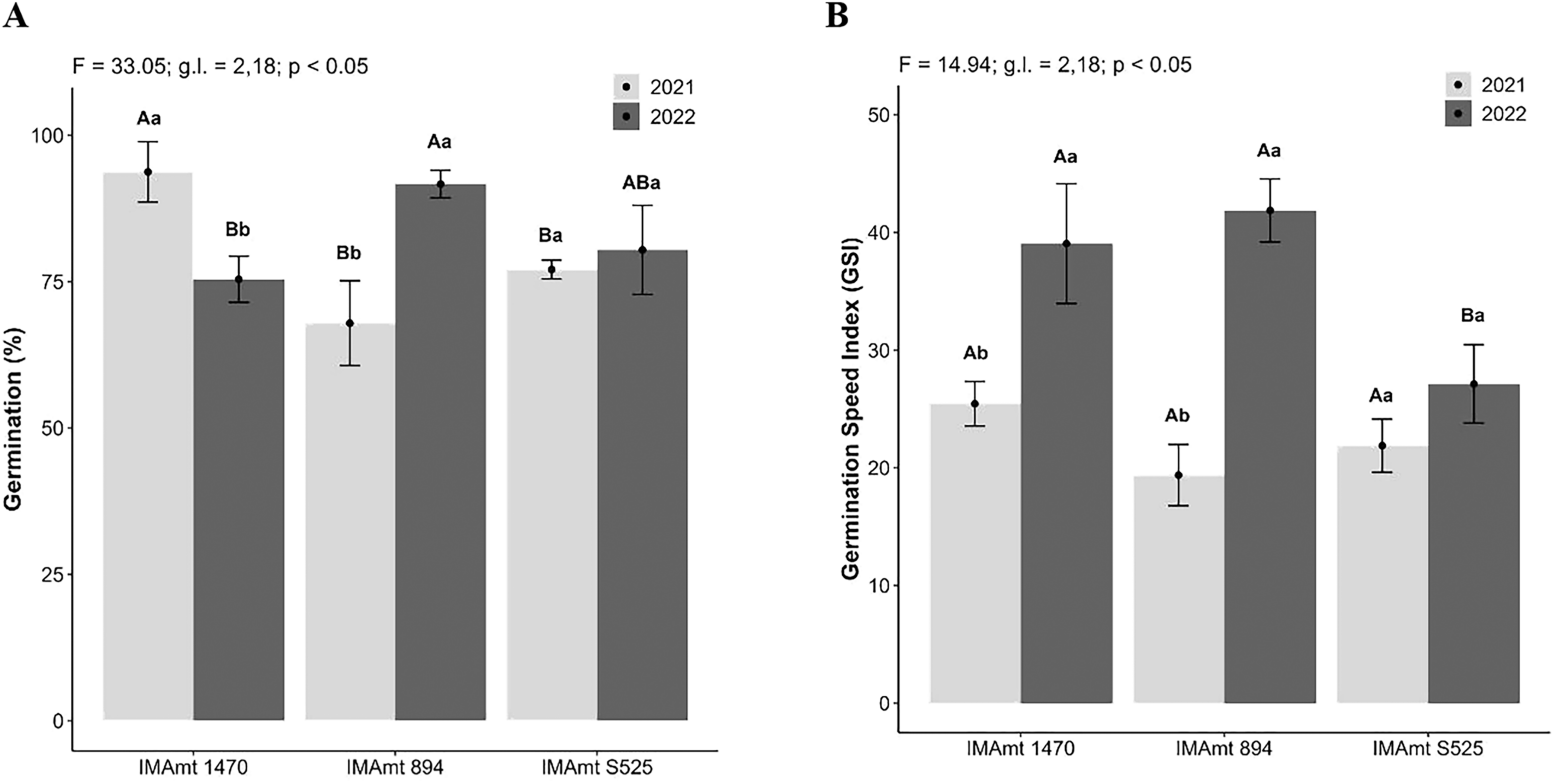
Germination (%) (A) and germination speed index (GSI) (B) of three safflower cultivars (IMAmt 1470, IMAmt 894, and IMAmt S525) in the two growing seasons (2021 and 2022). Different lowercase letters indicate *p* < 0.5 for cultivar between years, and different uppercase letters indicate *p* < 0.5 between cultivars.

There was a statistically significant difference between the cultivars for the GSI of the seeds harvested only in 2022, with IMAmt S525 exhibiting the lowest GSI, indicating slower germination compared to the other cultivars. For the year effect on the cultivars, IMAmt 1470 and IMAmt 894 showed the highest GSI in 2022, indicating faster germination and greater vigor (Figure 3B).

Values similar to those observed here in the 2021 crop for cultivar IMAmt 1470 were reported for cultivar NARI-6 with 91.43% and were higher than those obtained here for IMAmt S525 and IMAmt 894, for cultivar A-1 (86.27%) and cultivar NARI-57 (83.38%) by Gehlot et al.^52^ The values observed here for cultivar IMAmt 1470 are higher than those reported for soybeans by Pellizzari et al.,^53^ with a germination rate of 67%, by Brasil^29^ with reported value of 86%, and Guragain^54^ with a germination rate of 83%. The seeds produced by the cultivars IMAmt 1470 and IMAmt S525, in both years of cultivation, showed high germination vigor. For Asteraceae, the germination rate for high viability cultivars was higher than 76%.^55^ According to Schons et al.,^36^ one of the factors that influences the germination rate and physiological quality of seeds is the genotype, which varies between cultivars, as observed in this study.

The final germination rates of the seeds in storage were 95, 62, 99, 96, 83, 71, and 50% for the IMAmt 1470 cultivar at 0, 3, 6, 9.12, 15, and 18 months of storage, respectively. The IMAmt 894 cultivar showed rates of 68, 45, 81, 91, 78, 36, and 15% in 0, 3.6, 9.12, 15, and 18 months of storage, respectively. The IMAmt S525 cultivar showed 81, 45, 98, 83, 34, and 10% for 0, 3, 6, 9, 12, 15, and 18 months, respectively. According to Menegaes et al.,^56^ the vigor of safflower seeds can vary between cultivars and over the period they remain in storage, as observed in our study.

The storage time with the highest rate was 6 months for IMAmt 1470, which was 8% higher than IMAmt 894 and 1% higher than IMAmt S525, both at 6 months of storage for IMAmt S525 and at 9 months of storage for IMAmt 894. Overall, the highest viability of the safflower seeds is between 6 and 9 months of storage for all cultivars.

The standout cultivar (*p* < 0.5 associated with F: 5. 71 and g.l 12, 63 degrees of freedom), in terms of storage, was IMAmt 1470 with averages above 75% up to 12 months; for the other cultivars, similar percentages were observed between 60 and 90 days of storage (Figure 4A). A similar response was also seen for the GSI (Figure 3B).

**Figure 4:**
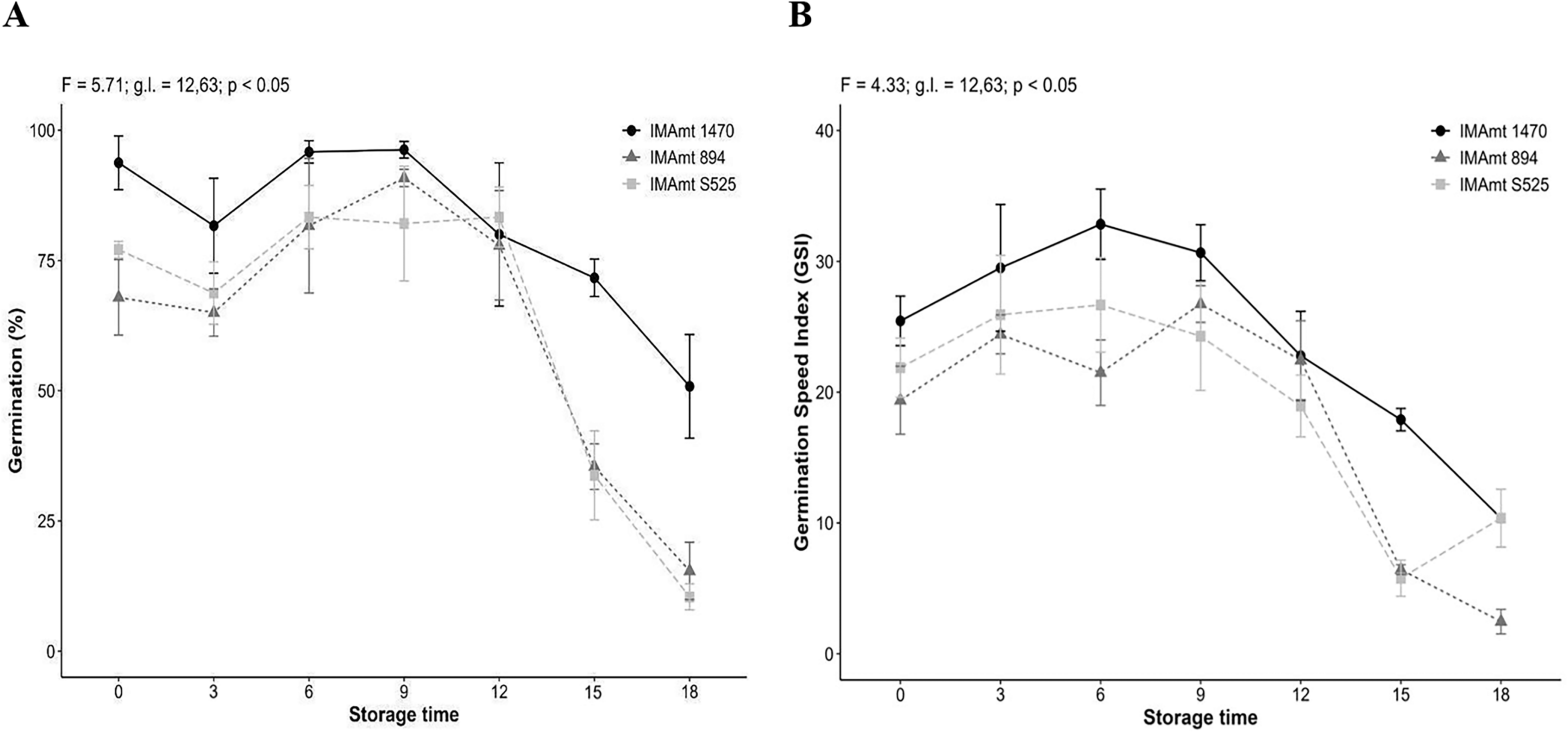
Germination (A) and germination speed index (GSI) (B) of safflower seed cultivars (IMAmt-1470, IMAmt-894, and IMAmt-S525) during aging.

A loss of physiological quality in safflower seeds was observed, with a decline in germination after four months of storage.^43^ This is different from what was observed in our study of safflower cultivars, as the decline in viability occurred after 12 months of storage. This loss of vigor, observed with reduced germination, is due to the natural aging of safflower seeds, as also observed by Tonguc et al.^57^ Since prolonged storage can lead to an accumulation of moisture in the seed, therefore, a reduction in germination, as pointed out by Oba et al.^44^ A longer storage period can promote the accumulation of moisture in the seeds, damaging its physiological quality.^42^ When compared to other oilseed crops such as peanuts and sesame, for example, the vigor of safflower seeds in our study is superior. The peanuts and sesame maintained seed vigor for 8 months of storage.^39^

Prolonging the viability in germination rate and vigor (GSI) of seeds in storage provides producers with security when planning plantings. Therefore, choosing safflower cultivars for production and use in various industrial sectors is more advantageous, as they maintain greater viability and vigor for a longer period and can be stored for up to a year. The selection of cultivars is a key factor when storing seeds for the long term.^30^ The most vigorous cultivars will be those with the highest GSI values and germination rates, as seen in this study for safflower (Figure 3) and in ref^58^ for soybeans, the main oilseed crop for biofuels.

### 3.3 Yield Parameters of the Three Safflower Cultivars

The safflower cultivars showed a high seed yield per hectare (SPH) of 6797.728 (kg ha^–1^) for IMAmt-1470 and 5245.720 (kg ha^–1^) for cultivar-S525 in the 2021 crop, with no statistical difference between the cultivars. In 2022, there was a drop in SPH of 73% for cultivar IMAmt-1470, 69% for IMAmt 894, and 16% for IMAmt S525, differing statistically from the other cultivars with the lowest reduction in SPH. When analyzing the effect of the year of cultivation on cultivars, a statistically significant difference was observed (*P* < 0.5), indicating that the year 2021 was favorable for all cultivars in terms of productivity. In terms of number of seeds per plant (NSP), values ranged from 597.634 (IMAmt 1470) to 846.812 (IMAmt 894), with no statistical difference between the cultivars in the 2021 harvest. However, when analyzing the 2022 harvest, it was observed that there was a reduction in NSP and a statistically significant difference for the IMAmt S525 cultivar, with values of 5245.720 in NSP, and a lower percentage reduction when compared to the other cultivars.

Yields can vary between crop genotypes, with lower values than those obtained here for the 2021 harvest for all cultivars and for the IMAmt S525 cultivar in the 2022 harvest are reported by Manvelian et al.,^48^ with yields of 2781 kg ha^–1^ ^47^; and 2448 kg ha^–1^,^24^ for the M12 cultivar, with a yield of 4,518 kg ha^–1^.

When looking at the productivity of other oilseed crops for energy purposes, such as sesame, Hasani et al.^59^ found values ranging from 591 to 812 kg ha^–1^ depending on the genotype and sunflower with a productivity of 2371 kg ha^–1^.^47^ A yield of 1,565.42 kg for safflower was obtained by Kamle et al.,^60^ which was close to that observed here for the year 2022, for the IMAmt1470 cultivar with the lowest SPH. When studying the safflower cultivars A-1 and NARI-6, Kamle et al.^61^ reported a more significant effect of the growing season than the cultivar effect, with an influence on both yield parameters and physiological development. This is in line with that observed in the present study, which was more significant for the safflower cultivars IMAmt 1470 and IMAmt 894 and influenced SPH in cultivar IMAmt S525, although with a reduction in SPH (16%).

The number of chapters per plant (NCP) is a marker of final productivity for safflower^62^ and can indicate more productive cultivars. The productive parameter NCP for this study showed that IMAmt 1470, IMAmt 894, and IMAmt S525 had averages of 34, 36, and 25, respectively, for 2021. In 2022, the IMAmt 1470, IMAmt 894m, and IMAmt S525 cultivars produced averages of 33, 10, and 25 NCP, respectively. Statistically, the safflower cultivars showed significant differences, with IMAmt 894 standing out as the most productive cultivar in terms of NCP, followed by IMAmt 1470. The effect of the year on the IMAmt 894 cultivar was also observed, with a 28% reduction when comparing 2022 and 2021, and was negatively affected by the year effect (Figure 5).

**Figure 5:**
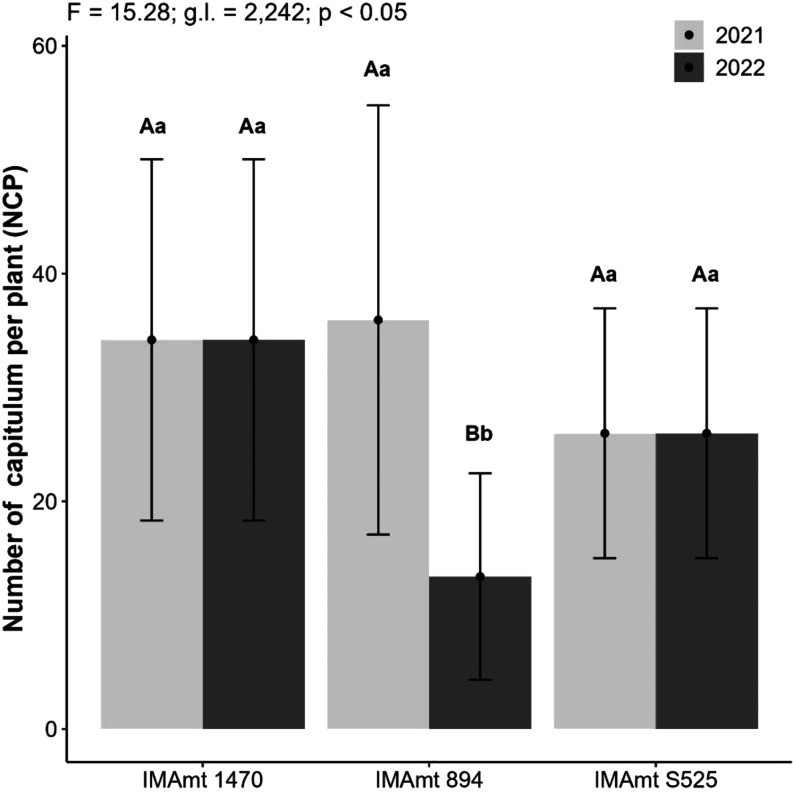
Number of chapters per plant (NCP) of the three safflower cultivars in two years of cultivation in 2021 and 2022.

The results found here are higher than those reported by Zanetti et al.^63^ for safflower (around 8.6 NCP). The main explanation could be due to the high number of branches, in which the chapters are formed and the greater number of these reflects on the high number of chapters per plant and final productivity,^64^ which has also been reported for FARAMIN safflower cultivars.^25^ The best yield performance is one of the factors that can indicate the safflower genotypes that are most tolerant to the soil and climate conditions of the planting region, as found in the study by Yeloojeh et al.^65^ for the Kouseh cultivars C116, C128, C4110, E2417, E2427, E2428, K12, K21, M420, S122, S149, and S3110. For the year effect, a different response to that observed in this study for NCP was recorded by Yeloojeh et al.,^24^ who obtained higher values of 18 to 33 and chapters per plant for safflower in the second crop in the year following the first due to the delay in planting. This may have also influenced the decrease in NCP for the IMAmt 894 cultivar.

Productive parameters such as NCP and NSC can be related to the final productivity of the crop, and, for safflower, it can vary depending on the cultivar studied.^66^ In NSC, the safflower cultivars IMAmt 1470, IMAmt 894, and IMAmt S525 presented averages of 17, 23, and 26, respectively, in 2021, with a reduction of 23% for IMAmt 1470, 26% for IMAmt 894, and 19% for IMAmt S525 in 2022 in NSC. The NSC was influenced by both the cultivar effect and the year of cultivation, showing a statistically significant difference (*p* < 0.5) in NSC between both cultivars and years of cultivation, with IMAmt S525 showing the highest, followed by IMAmt 894 (Figure 6).

**Figure 6:**
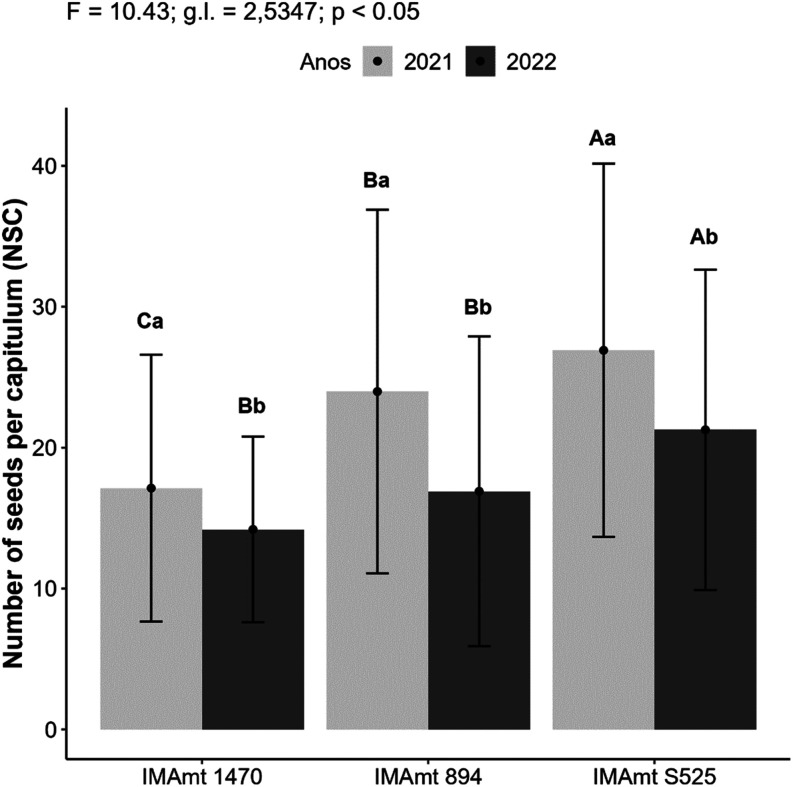
Number of seeds per chapter (NSC) of the three safflower cultivars in two years of cultivation in 2021 and 2022.

The year effect was stronger on NSC than the cultivar effect, with values of 29 to 39 NSC in the first year, and an increase of 30 to 44in the second year.^24^ This is in contrast to the results observed here for safflower cultivars, which showed higher NSC values in the first year of cultivation (Figure 5). Higher NSC values than those observed here were reported by Ebrahimian et al.^47^­(45.13) and were also observed in other oilseed crops. The yield parameters of soybean crops are also influenced by soil and climate conditions, cultivar, and growing season, being favored in milder periods.^67,68^ The variation in the results of the production parameters was also observed by Licata et al.^35^ when studying different safflower cultivars, a fact that may be related to the variations in environmental conditions between the years, such as the variation in humidity and temperature between the years of cultivation, influencing the cultivars.^69^

### 3.4 Percentage and Composition of Oil in Seeds of Three Safflower Cultivars

The cultivar with the highest seed oil yield was IMAmt S525, which differed statistically from the other cultivars (*p* < 0.5), with 35% representing 4 percentage points higher than cultivar IMAmt 1470 (31%) and 9% higher than cultivar IMAmt 894 (26%) for the 2021 crop year. In 2022, cultivar IMAmt 1470 showed an increase of 3%, and cultivar IMAmt 894 showed an increase of 6% in the concentration of oil in the seed; however, cultivar IMAmt S525 showed a reduction in the concentration of oil in its seeds by 4%, differing statistically from the other cultivars (*p* < 0.5). When the effect of the year on the concentration of oil in the seeds was analyzed, it was found that cultivars IMAmt 894 and IMAmt S525 differed statistically between the years 2021 and 2022, the former favoring 2022 and the latter 2021 (Figure 7).

**Figure 7:**
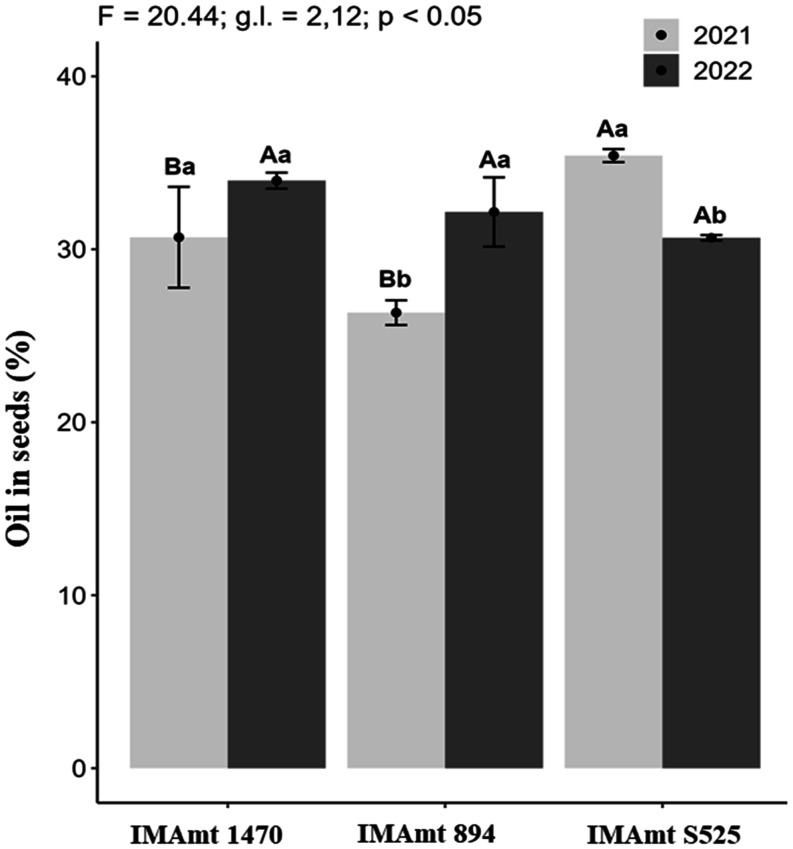
Oil yield in the seeds of three safflower cultivars in two years of cultivation (2021 and 2022). Different lowercase letters indicate *P* < 0.5 for cultivar between years, and different uppercase letters indicate *P* < 0.5 between cultivars.

The oil content of safflower cultivars varies depending on the cultivar.^24–26^ All the safflower cultivars analyzed here showed higher oil content values than the 21.8% reported for the crop in the studies by Ebrahimian et al.^47^ and for the Goldasht cultivar with 20.78%.^25^ Similar values obtained in this study were also reported for the safflower (32.49%) by Deviren et al.,^70^ and for cultivar NARI-57 (35.36%).^60^ When compared to other oilseed crops, safflower showed an oil content similar to that reported for sesame: 35.6% in the study by Ebrahimian et al.^47^ and lower than that obtained by Marcos-Filho,^50^ around 47.5% oil in sesame seeds. The IMAmt S525 cultivar can be classified as having a high oil content in the seeds, as it has values close to those indicated by Kadrivel et al.^26^ for the high oil content cultivars PBNS-12 and NARI-57, with 36.69% and 36.88% for cultivar Egypt 1,^71^ which are proximate to those observed in the present study.

The safflower oil from the cultivars analyzed here had the following fatty acids: linoleic, oleic, palmitic, and methyl esters. In the 2021 crop cycle, the majority of fatty acids observed for the cultivars were linoleic acid, followed by oleic and palmitic acids in all cultivars (Table 3). The cultivar with the highest percentage of linoleic fatty acid content for the 2021 growing season was the IMAmt 894 cultivar (71.82%), which was 1% higher than that observed for the IMAmt 1470 cultivar and 12.88% higher than that observed for the IMAmt S525 cultivar. In regard to oleic acid content, the IMAmt S525 cultivar showed the highest values, followed by IMAmt 1470 and IMAmt 894. In terms of palmitic acid content, the IMAmt 894 cultivar showed the highest, followed by IMAmt 1470 (Table 3). In the 2022 crop cycle, all the cultivars showed a decrease in the percentage of linoleic acid, with values of 23.82% for IMAmt 894. 20.5% for IMAmt 1470, and 4.04% for IMAmt S525, and an increase in the percentage of oleic acid. The cultivar with the highest percentage of linoleic acid was IMAmt S525, and for oleic acid, it was IMAmt 894 (Table 3), indicating their potential for biofuel production.

**Table 3:** Oil Profile of the Seeds of the Three Safflower Cultivars Grown in 2021 and 2022 (Expressed in %)

	year 2021			year 2022		
	IMAmt 1470	IMAmt 894	IMAmt S525	IMAmt 1470	IMAmt 894	IMAmt S525
palmitic acid	8.65	9.09	8.61	9.7	8.0	7.5
linoleic acid	70.80	71.82	58.94	50.3	48	54.9
oleic acid	15.45	14.38	28.03	36.4	41,1	34.9
methyl ester	5.10	4.71	4.42	3.5	2.9	2.6

The chemical composition of safflower oil observed in this work is in line with the literature, and the percentage of fatty acids varies depending on the cultivar^26^ with the majority being linoleic and oleic acids,^72^ which is also observed for soy,^73^ which is the main oilseed used to produce biofuels. The variation in the linoleic fatty acid content cited for the crop ranges from 8.99% for cultivar BC2F6-39-9-4,^74^ 17.9,^72^ and 56.4%,^70^ values lower than those obtained here (Table 3). Approximate percentages similar to those observed in this study for the cultivars IMAmt 894 and IMAmt 1470 were reported by Kurt et al.^71^ for the Iran 1 variety around 77.73%_.^75^ The percentages observed are close to those cited in the literature for *Sesamum indicum* L. (83%),^76,77^ soybeans (80%), linoleic fatty acid,^73^ and higher than 54.93% for the same crop^78^ and for canola (21.9%).^79,80^

The oleic fatty acid content attained for the cultivar is higher than that cited by Hou et al.^72^: 1% for the crop, and 7.91% for the Siena cultivar^25^; however, it was lower than that cited for the cultivars Montola-2000, Bhima, and BC2F6-39-9-4 (75.2%, 81.8%, 84.72%, respectively),^26^ which are considered to have a high oleic fatty acid content. The values for IMAmt S525, in the 2021 harvest and all the 2022 harvest (Table 3), are higher than those cited for spiny accessions of safflower (68%) and soybeans (20.44%).^78^ In the 2021 harvest, those obtained for cultivar IMAmt 894 are close to those reported for *Sesamum indicum* (50%)^47^ and canola (54%).^79^

The response observed for the physiological parameters of development and productivity analyzed here is consistent with that observed in the literature for both crops. One factor that can alter the oil profile of safflower cultivars is soil and the climatic conditions of cultivation linked to the sowing season^14,43,51,52,69,70^ and oilseeds such as sunflower.

The most suitable cultivars for field cultivation are those that stand out in terms of physiological development parameters, seed yield, and oil yield.^81^ Overall, the safflower cultivars that stick out for combining the greatest number of physiological, yield, oil content, and oil profile characters in sequence are IMAmt 1470, IMAmt 894, and IMAmt S525, the latter with less variation in the reduction of productive parameters remaining more stable between harvests and can be used for production in semiarid environments for the production of biofuels such as biodiesel and renewable hydrocarbons for the aviation sector.^82^

The MAmt 894, IMAmt S525, and IMAmt 1470 cultivars are suitable for cultivation as biomass to produce biofuels and renewable hydrocarbons, considering the combination of the highest number of physiological and productive parameters, seed yield, oil content, and profile. Relevant criteria to identify cultivars suitable for breeding programs focused on improved yield and oil content.^73^

In this context, the MAmt 894 cultivar stood out in eight parameters (SPH, NCP, NSC, and linoleic acid—harvest 2021; germination rate, IVG, and oleic acid—harvest 2022). The highest number of seeds, seeds per capitulum, and capitula per plant were found in the higher-yielding variety,^73^ as observed in our study.

IMAmt S525 stood out in seven parameters (PM, NSC, oil yield—harvest 2021; SPH, PM, NSP, NSC—crop year 2022) and IMAmt 1470 with six outstanding parameters (TG, germination and IVG in aging, NCP—crop year 2021; oil yield and oleic acid—crop year 2022). Environmental conditions influence production parameters, such as seed yield,^83^ oil, and oil profile, in oleic and linoleic acid concentrations,^84,85^ and seed quality, in which higher moisture provides lower physiological performance.^86,87^ Both environmental variations between similar seasons as well as daily variations in atmospheric conditions affect safflower cultivation.^88^ The higher temperature and solar postponement and lower atmospheric humidity have a positive influence on crop production parameters.^89^

All three cultivars have high standards of physiological quality, productivity, and oil profiles. The seeds can be stored for up to a year while maintaining high viability and germination vigor.

As safflower is not native to Brazil, this study represents a pioneering and essential effort toward understanding the development and productivity of safflower cultivars under Brazilian semiarid conditions. This study serves as a starting point for future large-scale commercial cultivation aimed at supplying various industrial sectors.

### 3.5 Industrial Applications of Safflower

Based on the data obtained and discussed in this study on phenological development, productivity, and oil profile parameters, cross-referenced with those cited in the literature, it is possible to highlight that safflower has the potential to meet the demand of seeds, biomass, and oil for various industrial sectors cited in the literature, such as biofuel, pharmacological/medical, food industry, and textile. In the biofuel sector, biodiesel, bioethanol, methane, and gasoline can be produced using biomass^90–92^ and biogas.^93^ The crop’s significant environmental contributions have low GHG emissions in its life cycle.^23,94,95^ In ref.^96^, 34 bioactive compounds have been classified as antioxidants, including^97^ quinolone C-glycosides and 2 alkaloids (hydroxy safflower yellow A, anhydrous safflower yellow B), with potential pharmacological/medical applications. Safflower oil can be used in chocolate production^98^ and in the textile industry using pigments found in their flowers.^99^

Further studies need to be carried out on the effect of atmospheric soil and climate conditions during the growing season, possibly using the integration of tools such as remote sensing to gain a more detailed understanding of the crop’s behavior during its phenological development.
